# Effect of Substrate Reduction Therapy in Comparison to Enzyme Replacement Therapy on Immune Aspects and Bone Involvement in Gaucher Disease

**DOI:** 10.3390/biom10040526

**Published:** 2020-03-31

**Authors:** Renuka P. Limgala, Ozlem Goker-Alpan

**Affiliations:** Lysosomal and Rare Disorders Research and Treatment Center (LDRTC), Fairfax, VA 22030, USA; ogoker-alpan@ldrtc.org

**Keywords:** Gaucher disease, bone involvement, enzyme replacement therapy, substrate reduction therapy, Osteoimmunology, RANK/RANKL, Osteopontin, MIP-1β

## Abstract

Gaucher disease (GD) is caused by mutations in the *GBA* gene, leading to deficient activity of the lysosomal enzyme glucocerebrosidase. Among all the symptoms across various organ systems, bone disease is a major concern as it causes high morbidity and reduces quality of life. Enzyme replacement therapy (ERT) is the most accepted treatment; however, there are still unmet needs. As an alternative, substrate reduction therapy (SRT) was developed using glucosylceramide synthase inhibitors. In the current study, the effects of ERT vs. SRT were compared, particularly the immunological and bone remodeling aspects. GD subjects were divided into three cohorts based on their treatment at initial visit: ERT, SRT, and untreated (UT). Immunophenotyping showed no significant immune cell alterations between the cohorts. Expression of RANK/RANKL/Osteoprotegerin pathway components on immune cells and the secreted markers of bone turnover were analyzed. In the ERT cohort, no significant changes were observed in RANK, RANKL or serum biomarkers. RANKL on T lymphocytes, Osteopontin and MIP-1β decreased with SRT treatment indicating probable reduction in osteoclast activity. Other secreted factors, Osteocalcin and RANKL/Osteoprotegerin did not change with the treatment status. Insights from the study highlight personalized differences between subjects and possible use of RANK pathway components as markers for bone disease progression.

## 1. Introduction

Gaucher disease (GD) (OMIM ID: 230800) is the most prevalent lysosomal disorder, caused by pathogenic mutations in the *GBA* gene, leading to a deficient activity of the lysosomal enzyme β-glucocerebrosidase (GCase). Deficiency of GCase results in the accumulation of glycosphingolipids in various organ systems, most notably in cells of mononuclear phagocyte system. The effects of the glycolipid accumulation are manifested in multiple organ systems, resulting in major signs and symptoms that include enlargement of the liver and spleen (hepatosplenomegaly), lung disease and skeletal abnormalities [1]. Among all these symptoms, bone disease is a major matter of concern for physicians as it causes high morbidity and reduces quality of life. The main clinical manifestations of skeletal disease in GD may be classified into a) bone marrow disease resulting in thrombocytopenia (low number of platelets) and anemia (reduced red blood cells) and b) structural involvement. Structural complications can further be subclassified into (1) focal infarcts leading to avascular necrosis (osteonecrosis), sclerosis and osteolytic lesions, (2) generalized osteoporosis and osteopenia, which result in reduced bone density and frequent fractures, and (3) local manifestations that include structural deformities (Erlenmeyer flask deformities) and cortical thinning [2]. Such extensive involvement of complications encompassing multiple facets of the skeletal system occurs in very few cases as the inherent pathology of a medical condition, but rather as a result of the response to external factors such as exposure to long-term corticosteroid medications, radiation therapy, organ transplants etc. This could indicate immune system alterations resulting from such factors may play a significant role in causing these bone complications. Bone is a mineralized connective tissue, which contains embedded osteocytes, and is covered by bone lining cells, osteoclasts, reversal cells and osteoblasts. Furthermore, bone is a living organ in continuous remodeling. Bone remodeling is a highly complex process of resorption by osteoclasts and matrix formation by osteoblasts. Osteoclasts are multinucleated cells that derive from the fusion of cells of monocyte/macrophage lineage under the influence of various molecular mediators [3]. The term osteoimmunology was coined many years ago to describe the research field that investigates the cross-regulation between skeletal and immune systems. Several immune cell subtypes including T/B lymphocytes and dendritic cells (DC) along with secreted factors participate in bone-immune system cross talk affecting osteoblast/osteoclast related bone remodeling [4,5].

Studies using animal models of GD have shown the involvement of osteoblasts in the bone pathophysiology of the disease [6]. Therefore, bone alterations observed in GD patients could be explained, at least partially, by changes in bone generating cells. On the other hand, it has been demonstrated that GCase deficiency is associated with increased osteoclastogenesis and bone resorption both in in vitro models and patients’ samples. In GD type 1, the number of cytotoxic T lymphocytes was found to be significantly lower in patients presenting bone involvement, and this correlated with higher levels of plasma tartrate resistant acid phosphatase (TRAP) activity, a putative marker of osteoclast cell activity [7,8,9]. Components of the RANKL/RANK/OPG pathway, consisting of the cytokine receptor activator of nuclear factor kappa-B ligand (RANKL), its signaling receptor, receptor activator of NF-κB (RANK), and the soluble decoy receptor osteoprotegerin (OPG) have been shown to be major effectors at multiple levels of the bone regeneration cycle and act as interfaces between immune and skeletal systems [10,11,12].

Macrophage-directed enzyme replacement therapy (ERT) has been the most accepted form of treatment for GD; however, there are still unmet needs in treating all aspects of the disease. As an alternative to ERT, substrate reduction therapy (SRT) was developed using glucosylceramide synthase inhibitors [13,14,15,16,17]. In the current internal review board (IRB) approved study (NCT02605603), we closely monitored and compared the effects of ERT vs. SRT, particularly the immunological aspects and secreted biomarkers involved in bone remodeling.

## 2. Materials and Methods

Subjects: Thirty-two patients with confirmed GD were enrolled into this active comparator study (NCT02605603). The handling of tissue samples and patient data was approved by the internal review board (Western IRB) including the procedure whereby all patients gave informed consent to participate in this study. Written informed consent was obtained using an IRB-approved informed consent form. At enrollment, a medical history was obtained and a detailed physical examination was performed. Medical records were reviewed as a part of the clinical evaluation and bone disease findings were assessed. All subjects were evaluated during three visits over a period of 12-18 months. Total subjects were divided into three cohorts based on their treatment at the time of initial visit: ERT, GD patients under long-term enzyme replacement therapy with velaglucerase alfa, (VPRIV^®^, Shire Human Genetic Therapies, Inc., MA, USA) or imiglucerase, (Cerezyme^®^, Sanofi Genzyme, Cambridge, MA) (n = 14, ERT-1 to ERT-14); SRT, GD patients who were switched to substrate reduction therapy with eliglustat (Cerdelga^®^, Sanofi Genzyme, Cambridge, MA, USA) during their first visit (n = 10, SRT-01-SRT-10); UT, GD subjects who were either untreated or had long interruption to treatments (n = 8, UT-01 to UT-08). Three subjects in the UT cohort (UT-01, UT-02 and UT-03) were started on SRT and one subject on ERT (UT-04) during subsequent visits. Only initial visit data from UT-07 were available as the subject discontinued participation and was not available for subsequent visits (Table 1).

Immunophenotyping: Direct immunofluorescence with specific antibodies was performed on peripheral blood as previously described [18,19] with some modifications using the following antibodies: anti-IgG1 FITC, anti-CD5-FITC, anti-CD8-FITC, anti-CD14-FITC, anti-CD22-FITC, anti-CD34-FITC, anti-IgG1-PE, anti-CD3-FITC/CD16^+^CD56-PE, anti-CD11C-PE, anti-CD21-PE, anti-CD27-PE, anti-CD183-PE, anti-CD194-PE, anti-CD20-PerCP and anti-HLA-DR-PerCP (BD Bioscience, San Jose, CA, USA). Anti-CD19-FITC, anti-IgA-FITC, anti-IgD-FITC, anti-CD8-PE, anti-CD19-PE, anti-IgG-PE, anti-IgG1-PerCP, anti-CD3-PerCP, anti-CD4-PerCP, anti-CD8-PerCP and anti-CD3-APC (Invitrogen, Carlsbad, CA, USA). Anti-Lineage-FITC (anti CD3/CD14/CD16/CD19/CD20/CD56), anti-CD196-PerCP, anti-IgM-PerCP and anti-CD45-APC (Biolegend, San Diego, CA, USA). Anti-CD4-FITC (eBioscience, San Diego, CA, USA), anti-CD45RO-FITC (Abcam, Cambridge, MA, USA) and anti-BDCA2-APC (Miltenyi Biotech, San Diego, CA, USA). Briefly, after washing the whole blood with PBS, 100 µl of blood was stained with the relevant cocktail of antibodies at 4 °C for 30 min followed by red blood cell lysis using BD FACS lysis solution (BD Bioscience, San Jose, CA, USA). Samples were acquired on Accuri C6 flow cytometer (BD Bioscience, San Jose, CA, USA) and analyzed using FCS express software (De Novo software, Glendale, CA, USA). During acquisition, a lymphocyte gate was assigned and 10,000 events were collected for the T cells and NK cells, and 25,000 events for the B cell analysis. For dendritic cells, a million ungated events were acquired.

Assessment of Bone biomarkers: Plasma samples were collected by centrifuging the whole blood within 12 h of collection and stored at −20 °C till use. Enzyme linked immunosorbent assays (ELISAs) were carried out using plasma samples for secreted factors—RANKL, OPG (Origene technologies, Rockville, MD, USA), osteocalcin, osteopontin, MIP-1β and CCL18 (Thermo Fisher Scientific, Waltham, MA, USA)—according to manufacturers’ protocols.

Statistical analysis: All statistical analysis was performed using GraphPad Prism software (GraphPad Software, Inc., La Jolla, CA, USA) and graphs were generated as dot plots. Statistical evaluation of differences was performed using Wilcoxon signed-rank test for comparing results between visits for each cohort. P-values were indicated where found significant, *: p < 0.05; **: p < 0.01.

## 3. Results

Bone involvement was assessed with the presence of bone pain, pathological fractures, bone density and radiological changes (Erlenmeyer flask and cystic deformities), bone marrow infiltration, history of osteonecrosis and previous surgery (Table 2). At enrollment, 72% of subjects presented with radiological changes (23/32), and 59% reported bone pain (19/32). Five subjects had severe skeletal involvement (three in the SRT and two in the ERT cohort), and all of these were splenectomized. Among the subjects who had a history of osteonecrosis, half of them were splenectomized (4/8). There were eight untreated patients at presentation, and three with moderate to severe bone disease were started on SRT, and one was treated with ERT. All subjects had involvement of the bone marrow. Bone density changes were observed about 50% of the total subjects (16/32), and there were five subjects with osteoporosis in the SRT, six subjects in the ERT cohorts, and three among the untreated. Overall, skeletal manifestations were equally represented in each treatment group on presentation.

**Figure 1:**
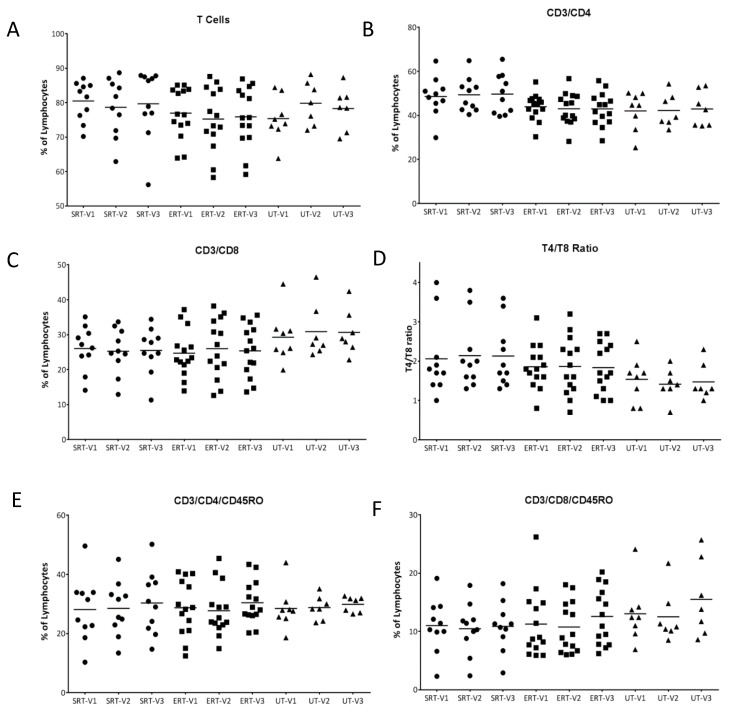
T-lymphocytes and subsets in GD patients. Percentages of T-lymphocytes (CD3+) from peripheral blood of GD patients at each visit were assessed using flow cytometry and plotted (**A**). T helper cells (CD3+/CD4+) (**B**) and cytotoxic T cells (CD3+/CD8+) (**C**) were calculated and a ratio of CD4 to CD8 cells was plotted (**D**). Similarly memory subsets of CD4 (CD3+/CD4+/CD45RO+) and CD8 T cells (CD3+/CD8+/CD45RO+) were calculated and plotted (**E**,**F**).

At every visit, after the clinical evaluation of each subject, peripheral blood was drawn for in-depth immunophenotyping. Flow cytometry-based immune profiling analysis was performed to elaborate on T/B-lymphocytes, NK/NKT cells and dendritic cell fractions in peripheral blood. Overall percentages of T lymphocytes, T helper cells, and cytotoxic T cells were maintained between visits within each cohort. However, cytotoxic T cells were found to be higher in the UT cohort resulting in lower T helper to cytotoxic T cell ratio. In subjects UT-02, UT-03 and UT-04, an increase in T helper to cytotoxic T cell ratio was observed, most likely as a result of the initiation of treatment. When memory T cell subsets were analyzed using CD45RO, no significant differences were observed either between cohorts or between visits within each cohort (Figure 1A–F).

**Figure 2:**
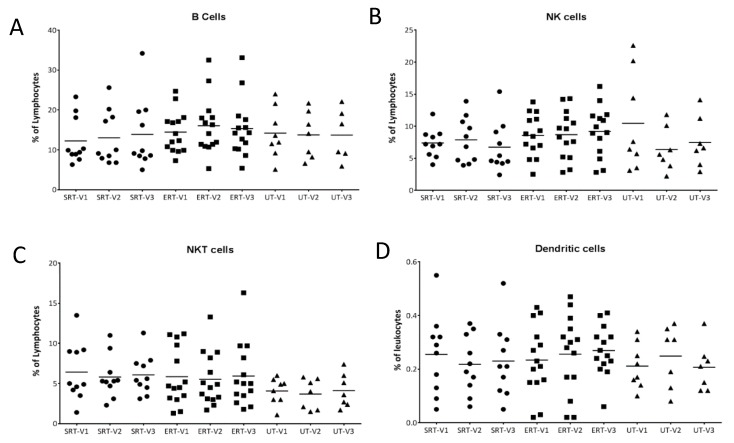
B-lymphocytes, NK, NKT and dendritic cells in GD patients. Percentage of B-lymphocytes (CD20+), NK cells (CD3-/CD16+ or CD56+), NKT cells (CD3+/CD16+ or CD56+) from peripheral blood of GD patients at each visit were assessed using flow cytometry and plotted (**A**–**C**). Dendritic cells were enumerated as Lin-/CD34-/HLA DR+ cells and plotted as a percentage of total leukocytes (**D**). • SRT cohort; ■ ERT cohort; ▲ UT cohort.

Other immune cell types, namely B lymphocytes, NK cells, NKT cells and dendritic cells, were quantified at each visit but no significant differences were found, indicating that treatment differences did not influence overall immune cell subsets in a significant manner (Figure 2A–D).

**Figure 3:**
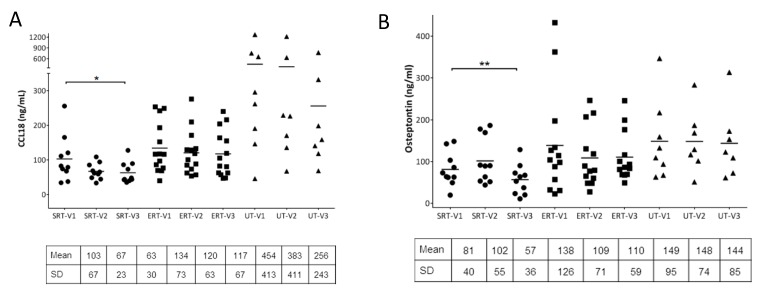

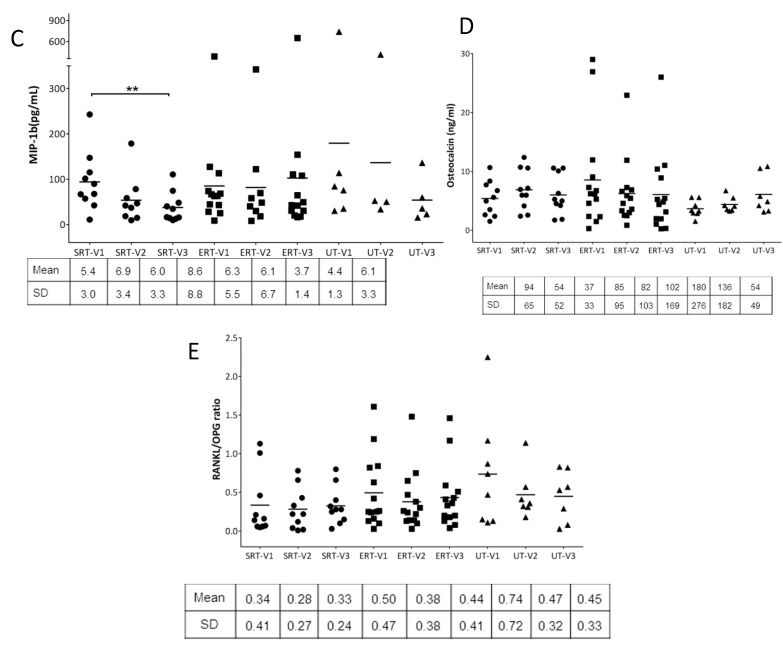
**Analysis of secreted biomarkers from plasma:** Macrophage activation marker, CCL18 was quantitated using ELISA from plasma samples at each visit and compared (**A**). Bone recycling biomarkers, osteopontin, osteocalcin, and bone disease markers, MIP-1β were quantified at each visit and plotted (**B**–**D**). Secreted RANKL and Osteoprotegerin (OPG) were quantified from plasma were plotted as RANKL to OPG ratio at each visit (**E**). Paired student’s t-test was performed to calculate significance values and included in the plots where significant difference was observed. *: p < 0.05; **: p < 0.01. Mean and +/− SD values are noted below individual plots. • SRT cohort; ■ ERT cohort; ▲ UT cohort.

In GD patients, chemokine (C-C motif) ligand 18 (CCL18) is known to be secreted at elevated levels by activated macrophages and has been used as biomarker to study response to ERT. CCL18 was measured using ELISA from the plasma samples collected from all the subjects during each visit. As expected, CCL18 levels were markedly elevated at visit 1 in the UT cohort compared to the SRT and ERT (p value = 0.008 and 0.02 respectively). No significant differences were observed within the ERT cohort between visits as expected. In subjects UT-01 to UT-04 who had started treatment for GD, there was a decrease in CCL18 levels between visits 2 and 3 compared to visit 1 as a positive response to therapy. Interestingly, in the SRT cohort, subjects showed overall lower CCL18 in subsequent visits compared to visit 1 (p value = 0.027) (Figure 3A).

Osteopontin (OPN) and osteocalcin (OC) are major non-collagenous proteins that play vital roles in bone remodeling and homeostasis. OPN is a multifunctional protein which promotes osteoclastogenesis and osteoclast activity as well as osteoclast survival and motility. OPN is also considered an atypical immune regulator and higher levels of OPN have been associated with inflammatory bone diseases. When OPN was measured from plasma samples from each cohort during multiple visits, OPN levels did not change between visits for the ERT and UT cohorts. In the SRT cohort, while OPN was found to be slightly elevated in seven out of 10 subjects, in all the subjects OPN levels were reduced by visit 3, especially compared to visit 1 (p value = 0.009) (Figure 3B). Osteocalcin, which is produced by osteoblasts, is widely regarded as a marker of bone formation and plasma concentrations of osteocalcin are used as markers of bone formation. In the current study, no significant differences in plasma OC levels were observed within the SRT and ERT cohort between visits. However, within the UT cohort, three UT subjects (UT-01, UT-03 and UT-04) showed increase in OC by visit 3 compared to visit 1, most likely as a result of treatment initiation (Figure 3C). Macrophage inflammatory protein-1 beta (MIP-1β) has been shown to be a marker for bone disease in GD [20]. Secreted MIP-1β level was significantly decreased by visit 3 in the SRT cohort compared to visit 1 (p value = 0.004) (Figure 3D). RANK/RANKL/OPG signaling has been shown to be critical in osteoclastogenesis and influencing bone remodeling. Serum concentrations of RANKL and OPG were analyzed for each subject at every visit and expressed as RANKL/OPG ratio. There were significant differences in RANKL/OPG ratio within the ERT cohort between visits. In the SRT cohort, four out of 10 subjects showed lower RANKL/OPG ratios due to an increase in OPG concentration. Within the UT cohort, three UT subjects (UT-01, UT-03 and UT-04) showed a decrease in RANKL/OPG ratio by visit 3 compared to visit 1, again as a likely result of treatment initiation (Figure 3E).

**Figure 4:**
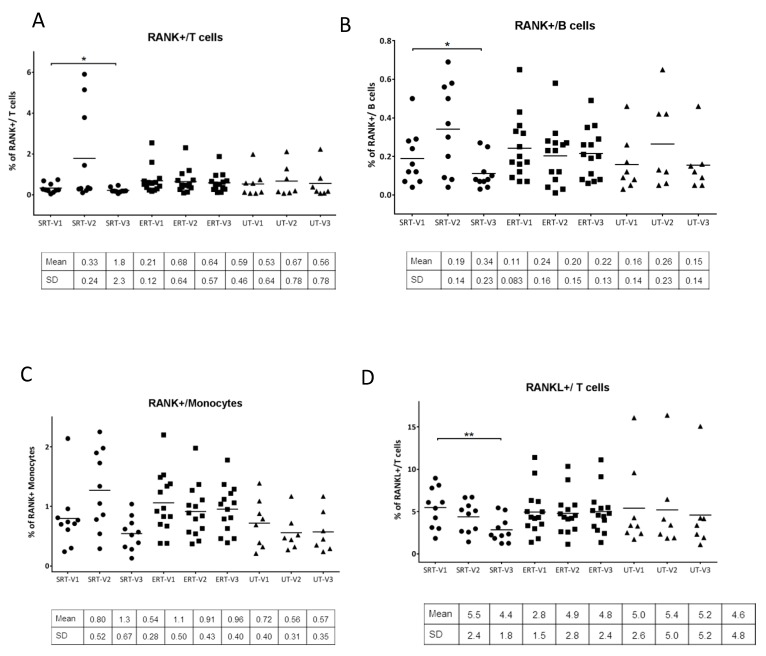
Analysis of RANK and RANKL on immune cell subsets: Percentage of RANK+ T-lymphocytes (CD45+/CD3+/RANK+), B-lymphocytes (CD45+/CD20+/RANK+) and monocytes (CD45+/CD14+/RANK+) and RANKL+ T-lymphocytes (CD45+/CD3+/RANKL+) at each visit quantitated using flow cytometry analysis from peripheral blood samples at each visit and compared (A-D). Paired student’s t-test was performed to calculate significance values and included in the plots where significant difference was observed. *: p < 0.05; **: p < 0.01. Mean and +/− SD values are noted below individual plots. • SRT cohort; ■ ERT cohort; ▲ UT cohort.

Cell surface expression of RANK on T and B lymphocytes and monocytes as well as RANKL expression on T lymphocytes was assessed using flow cytometry. In subjects within the ERT cohort, no major alterations were observed in surface expression of RANK or RANKL over the three visits. Within the SRT cohort, there was a marked increase at visit 2 in the expression of RANK on T cells and B cells (in four out of 10 subjects), and monocytes (in five out of 10 subjects). However, these alterations were seen to be reversed by visit 3. Overall cell surface expression of RANK was in fact significantly reduced on both T and B lymphocytes (p value = 0.019 and 0.012 respectively) at visit 3 compared to visit 1. RANKL expression on T lymphocytes at visit 3 was also significantly reduced in the SRT cohort compared to visit 1 (p value = 0.002) (Figure 4A–D).

## 4. Discussion

While GD affects multiple organ systems, skeletal complications are a common occurrence in majority of GD patients with debilitating consequences. Skeletal system involvement in GD includes growth retardation, structural distortions like Erlenmeyer flask deformities, lytic lesions, avascular necrosis, osteopenia, osteoporosis, bone marrow infiltration and bone crises. GD patients are also susceptible to fractures and bone surgery at higher rates. Furthermore, the severity of bone manifestations does not necessarily reflect the pathological involvement in other organ systems [2]. Such a wide array of complications in skeletal system points to a complex inter-linking of multiple pathways in bone development and bone remodeling over a time period. Since the hallmark of GD is the accumulation of sphingolipids including GL-1 and lyso GL-1, especially in the cells of monocyte/macrophage lineage, the effect of immune system modulation on bone development and remodeling is not trivial. It has been shown that although ERT is associated with improvement of bone crises and other bone complications over a period of time, not all symptoms are reversible. Moreover, it has been shown that certain GD patients continue to develop avascular necrosis (AVN) even after being on long-term ERT, especially if the treatment was delayed more than 2 y after diagnosis [21]. SRT was developed as alternate mode of treatment in adult GD patients with an aim to reduce accumulating glycosphingolipids by inhibiting their synthesis. SRT consists of small compounds that are taken orally and have the potential to rapidly diffuse into various tissues, with potential advantages of better prevention of bone complications due to drug delivery in the bone compartments. While certain components of bone disease see improvement with SRT, it is not possible to conclude with certainty as to superiority of SRT over ERT regarding bone complications [15,16,17,22,23,24]. Recent case reports demonstrate that judicious use of the SRT-ERT combination therapy may benefit in certain GD patients [25,26].

The skeletal system is a highly complex and dynamic structure with continuous turnover as a result of its constant remodeling cycle, essential for maintaining the integrity of the skeleton. Bone remodeling is a lifelong process consisting of three consecutive phases: bone resorption, during which osteoclasts digest the old bone; reversal, when mononuclear cells appear on the bone surface; and formation or ossification, when osteoblasts lay down new bone until the resorbed bone is completely replaced. In normal bone remodeling, there is no net change in bone mass and strength. However, the bone remodeling cycle may be derailed at multiple points, resulting in metabolic bone disorders [12]. In the current study, we investigated the influence of peripheral immune system on bone remodeling and whether bone remodeling defects could be assessed using the secreted markers of bone turnover including soluble RANKL (sRANKL), OPG, OPN, OC and MIP-1β. In addition, the expression of RANK/RANKL pathway components on relevant immune cell types was analyzed using flow cytometry. The results were then followed with their treatment status over a period of time. When major immune subsets were compared within the three cohorts, no significant differences were observed in overall fraction of lymphocytes, NK/NKT cells or dendritic cells. A decrease in the concentration of the chemokine CCL18 following SRT treatment in the SRT and UT cohorts indicated a reduction in macrophage inflammation in the subjects. Another cytokine, OPN, is known to play multiple roles in bone remodeling and acts as a positive regulator of osteoclastogenesis. It has also been indicated that OPN could be a bridge between bone and the immune system. In the SRT cohort, OPN and MIP-1β concentrations were found to be significantly lower at visit 3, indicating a probable reduction in osteoclast activity that could result in lower bone resorption. Other secreted factors, OC and RANKL/OPG did not change over time with the treatment status.

RANK is a member of the TNF family and its ligand, RANKL, was initially identified to be expressed on T cells and enhance dendritic cell survival. Later it was shown that RANKL-expressing T cells can also activate RANK-expressing osteoclasts, thereby mimicking RANKL-expressing osteoblasts and explaining the crosstalk of immune cells and bone. This crosstalk was found to be a causative factor for the bone loss observed in patients with a chronically activated immune system which induces osteoclastogenesis, thereby shifting the balance in favor of bone resorption over bone deposition [3,10,11,27]. In order to study the expression of RANK and RANKL on immune cell subsets, cell surface expression of these molecules was studied using flow cytometry. In a subset of the SRT cohort, there was a marked increase in expression of RANK on T and B lymphocytes as well as monocytes (Figure 4A–C). This effect was not only reversed by visit 3, but the overall RANK expression was reduced in the whole cohort. The results indicate that the cell-surface changes observed in the SRT cohort at visit 2 could be a result of initial reaction to the SRT treatment which did not have long term effects as seen by normalization or improvement by visit 3. RANKL expression on T cells was also significantly reduced in the SRT cohort by visit 3. Taken together these results indicate the reduction in osteoclastogenic biomarkers in the SRT cohort compared to the ERT cohort. One of the limitations of the study was inclusion of a higher ratio of females to males (24F/8M) which resulted from unbiased recruiting given our center’s patient population. However, the distribution of bone disease was comparable between females and males in the study subjects as seen in Table 1 and Table 2. Another limitation of the study was the brief period of study, as a result of which the correlation of the findings to the improvement of clinical symptoms has not been performed since that requires a longer follow up. However, insights from the study highlight personalized differences between subjects with different treatment modalities.

## Figures and Tables

**Table 1:** Basic characteristics of the subjects. Pathogenic mutations in *GBA* gene, treatment at initial and follow up visits are noted. M, Male; F, female; ERT, enzyme replacement therapy; SRT, Substrate reduction therapy; UT, Untreated. ERT ^¶^ -ERT with velaglucerase alfa (VPRIV^®^), ERT * - ERT with imiglucerase (Cerezyme^®^), SRT- eliglustat (Cerdelga^®^).

ID	Gender	Age (Years)	Genotype	Initial Visit	Follow-Up Visit
SRT-01	F	50	N370S/N370S	ERT ^¶^	SRT
SRT-02	M	59	N370S/N370S	ERT *	SRT
SRT-03	F	46	N370S/N370S	ERT *	SRT
SRT-04	F	57	N370S/N370S	ERT *	SRT
SRT-05	F	35	N370S/R463C	ERT *	SRT
SRT-06	F	37	N370S/R463C	ERT *	SRT
SRT-07	F	24	N370S/L444P	ERT *	SRT
SRT-08	F	62	N370S/R463C	ERT *	SRT
SRT-09	F	52	1448C/L444P	ERT ^¶^	SRT
SRT-10	F	47	N370S/N370S	ERT ^¶^	SRT
ERT-01	F	34	N370S/L444P	ERT ^¶^	ERT ^¶^
ERT-02	F	35	N370S/R120Q	ERT ^¶^	ERT ^¶^
ERT-03	F	45	N370S/N370S	ERT ^¶^	ERT ^¶^
ERT-04	F	61	N370S/L444P	ERT ^¶^	ERT ^¶^
ERT-05	F	20	L444P/L444P	ERT *	ERT *
ERT-06	M	18	L444P/L444P	ERT *	ERT *
ERT-07	F	10	L444P/L444P	ERT *	ERT *
ERT-08	F	27	N370S/L444P	ERT *	ERT *
ERT-09	F	42	N370S/L444P	ERT *	ERT *
ERT-10	M	50	N370S/L444P	ERT ^¶^	ERT ^¶^
ERT-11	M	76	N370S/N370S	ERT *	ERT *
ERT-12	M	14	L444P/L444P	ERT *	ERT *
ERT-13	F	40	L444P/R463C	ERT ^¶^	ERT ^¶^
ERT-14	F	23	N370S/W381X	ERT ^¶^	ERT ^¶^
UT-01	M	27	C677T/C677T	UT	SRT
UT-02	F	56	N370S/N370S	UT	SRT
UT-03	M	38	N370S/N370S	UT	SRT
UT-04	M	34	N370S/N370S	UT	ERT ^¶^
UT-05	F	36	N370S/N370S	UT	UT
UT-06	F	32	N370S/N370S	UT	UT
UT-07	F	61	N370S/N370S	UT	UT
UT-08	F	58	N370S/N370S	UT	UT

**Table 2:** Bone disease in subjects at the time of enrollment. Bone involvement for each subject is characterized by bone marrow infiltration, bone pain, EM-flask deformity, cystic/lytic lesions, osteoporosis, osteopenia and avascular necrosis. Subjects who have undergone splenectomy are indicated.

ID	Splenectomy	Bone Surgery	Bone Pain	Bone Marrow Infiltration	EM-Flask Deformity	Cystic/ Lytic Lesions	Pathologic Fractures	Osteo Penia	Osteo Porosis	AVN
SRT-01	No	No	Moderate	Moderate dark marrow	No	No	No	No	No	No
SRT-02	No	No	Moderate	Patchy dark marrow	No	Yes	No	No	Yes	No
SRT-03	No	No	Moderate	Patchy dark marrow	Yes	No	No	Yes	No	No
SRT-04	No	No	Mild	Patchy dark marrow	Yes	Yes	No	No	Yes	No
SRT-05	No	No	Mild	Mild symmetric dark marrow	No	No	No	Yes	No	No
SRT-06	No	No	Mild	Mild symmetric dark marrow	No	No	No	No	No	No
SRT-07	No	No	No	Patchy dark marrow	Yes	No	No	No	No	No
SRT-08	Yes	Yes	Moderate	Extensive dark marrow	Yes	Yes	Yes	Yes	Yes	Yes
SRT-09	Yes	Yes	Moderate	Mild symmetric dark marrow	Yes	Yes	Yes	Yes	Yes	No
SRT-10	Yes	Yes	Severe	Marrow infarcts	Yes	Yes	No	Yes	Yes	Yes
ERT-01	No	No	No	Mild patchy dark marrow	Yes	No	No	No	No	No
ERT-02	No	No	Moderate	Patchy, hypo intense marrow	Yes	No	No	Yes	No	No
ERT-03	Yes	Yes	No	Heterogeneous dark marrow	Yes	Yes	No	Yes	Yes	No
ERT-04	Yes	Yes	Moderate	Marrow infarcts/patchy dark	Yes	Yes	Yes	Yes	Yes	Yes
ERT-05	No	No	No	Patchy dark marrow	Yes	No	No	No	No	No
ERT-06	No	No	No	Patchy dark marrow	No	No	No	No	No	No
ERT-07	No	No	No	Patchy dark marrow	No	No	No	No	No	No
ERT-08	No	No	No	Patchy dark marrow	Yes	No	No	Yes	Yes	No
ERT-09	No	Yes	Moderate	Marrow infarcts	Yes	Yes	No	Yes	No	Yes
ERT-10	No	No	No	Marrow infarcts	No	No	No	Yes	Yes	Yes
ERT-11	No	No	Moderate	Marrow infarcts	Yes	Yes	No	Yes	Yes	Yes
ERT-12	No	No	Moderate	Patchy dark marrow	Yes	No	No	No	No	No
ERT-13	Yes	Yes	Severe	Marrow infarcts	Yes	Yes	No	Yes	Yes	Yes
ERT-14	No	No	Mild	Symmetric dark marrow	Yes	No	No	Yes	No	No
UT-01	No	No	No	Patchy dark marrow	No	No	No	No	No	No
UT-02	No	Yes	Moderate	Marrow infarcts/dark marrow	Yes	No	No	Yes	No	Yes
UT-03	No	No	No	Confluent dark marrow	Yes	No	No	No	Yes	No
UT-04	Yes	No	No	Patchy dark marrow	Yes	No	No	No	No	No
UT-05	No	No	No	Symmetric dark marrow	Yes	No	No	No	Yes	No
UT-06	No	No	No	Symmetric dark marrow	Yes	No	No	Yes	No	No
UT-07	No	No	Moderate	Mild symmetric dark marrow	No	No	No	Yes	No	No
UT-08	No	No	Mild	Patchy dark marrow	Yes	No	No	Yes	Yes	No
